# Twin Screw Melt Granulation of Simvastatin: Drug Solubility and Dissolution Rate Enhancement Using Polymer Blends

**DOI:** 10.3390/pharmaceutics16121630

**Published:** 2024-12-23

**Authors:** Rasha M. Elkanayati, Indrajeet Karnik, Prateek Uttreja, Nagarjuna Narala, Sateesh Kumar Vemula, Krizia Karry, Michael A. Repka

**Affiliations:** 1 Department of Pharmaceutics and Drug Delivery, School of Pharmacy, The University of Mississippi, Oxford, MS 38677, USA; rmelkana@go.olemiss.edu (R.M.E.); puttreja@go.olemiss.edu (P.U.); nnarala@go.olemiss.edu (N.N.); svemula@olemiss.edu (S.K.V.); 2 BASF Corporation, Pharma Solutions, Tarrytown, NY 10591, USA; krizia.karry@basf.com; 3 Pii Center for Pharmaceutical Technology, The University of Mississippi, Oxford, MS 38677, USA

**Keywords:** twin screw melt granulation, hot-melt extrusion, amorphous solid dispersion, Soluplus, Kollicoat IR, solubility enhancement, miscibility, poorly water-soluble drug

## Abstract

**Background/Objectives:** This study evaluates the efficacy of twin screw melt granulation (TSMG), and hot-melt extrusion (HME) techniques in enhancing the solubility and dissolution of simvastatin (SIM), a poorly water-soluble drug with low bioavailability. Additionally, the study explores the impact of binary polymer blends on the drug’s miscibility, solubility, and in vitro release profile. **Methods:** SIM was processed with various polymeric combinations at a 30% *w*/*w* drug load, and a 1:1 ratio of binary polymer blends, including Soluplus^®^ (SOP), Kollidon^®^ K12 (K12), Kollidon^®^ VA64 (KVA), and Kollicoat^®^ IR (KIR). The solid dispersions were characterized using modulated differential scanning calorimetry (M-DSC), powder X-ray diffraction (PXRD), and Fourier-transform infrared spectroscopy (FTIR). Dissolution studies compared the developed formulations against a marketed product. **Results:** The SIM-SOP/KIR blend showed the highest solubility (34 µg/mL), achieving an approximately 5.5-fold enhancement over the pure drug. Dissolution studies showed that SIM-SOP/KIR formulations had significantly higher release profiles than the physical mixture (PM) and pure drug (*p* < 0.01). Additionally, their release was similar to a marketed formulation, with 100% drug release within 30 min. In contrast, the SIM-K12/KIR formulation exhibited strong miscibility, but limited solubility and slower release rates, suggesting that high miscibility does not necessarily correlate with improved solubility. **Conclusions:** This study demonstrates the effectiveness of TSMG, and HME as effective continuous manufacturing technologies for improving the therapeutic efficacy of poorly water-soluble drugs. It also emphasizes the complexity of polymer–drug interactions and the necessity of carefully selecting compatible polymers to optimize the quality and performance of pharmaceutical formulations.

## 1. Introduction

Simvastatin (SIM) is an active pharmaceutical ingredient (API) from the statin class that is well known for its anti-hyperlipidemic activity. As per the drug product labeling, it is available as 5 mg, 10 mg, 20 mg, 40 mg, and 80 mg strengths; however, the usual recommended dose for adults is 20 mg to 40 mg taken once daily in the evening [1]. It is synthesized as a prodrug with an inactive lactone ring. Following oral administration, it undergoes metabolism in the liver and intestinal mucosa by cytochrome-3A to yield the active β-dihydroxy acid form [2]. SIM and its metabolites are potent inhibitors of 3-hydroxy-3-methyl-glutaryl-coenzyme A (HMG-CoA) reductase. This enzyme catalyzes the conversion of HMG-CoA to mevalonate, which is a rate-limiting step in cholesterol biosynthesis. Consequently, the enzyme inhibition leads to an upregulation of low-density lipoprotein (LDL) receptors, promoting the uptake of LDL-cholesterol (LDL-C) from blood to the liver and hence causes a reduction in plasma LDL-C and total cholesterol [3,4]. SIM is a white, crystalline, and non-hygroscopic compound with a molecular weight of 418.6 g/mol. Simvastatin’s solubility in water greatly varies in the literature, with reported values of 1.45 μg/mL [3], 2.23 μg/mL [5], 10 μg/mL [6], and a maximum of 30 μg/mL [7]. Even at the highest reported solubility, a dose strength of 10 mg or higher would classify SIM under Class II of the Biopharmaceutics Classification System (BCS). The water insolubility limits gastrointestinal absorption; moreover, the active metabolite β-hydroxy acid undergoes extensive first-pass metabolism in the liver, resulting in a half-life of approximately 1.9 h and a bioavailability of less than 5% [8,9,10].

Solid dispersion (SD) has been shown to be a successful approach for improving solubility of many APIs [3,8,11]. This technique involves dispersing one or more active drugs into an inert carrier matrix in the solid state. This can be achieved through various methods, including hot-melt extrusion (HME), spray drying, freeze drying, supercritical anti-solvent techniques, co-precipitation, solvent evaporation, fusion, and electrostatic spinning [11]. SDs of simvastatin (SIM) have been developed using various methods, as documented in the literature. The most recent approaches are summarized in Table 1.

Hot-melt extrusion (HME) is a continuous, cost effective, and easily scalable technology from the pilot to the industrial scale [21,22]. The technique employs shear and heat to mix API and polymers to obtain filaments, films, and granules [23,24]. Furthermore, HME is solvent free, which distinguishes it from solvent-dependent methods commonly used in solid dispersion preparations [25,26]. The absence of organic solvents eliminates the need for additional drying steps, reducing manufacturing costs and mitigating the risk of residual solvent toxicity [27,28]. On the other hand, this method has some limitations, including the possible thermal degradation of the drug.

In addition to HME, an extruder can be effectively utilized for a granulation technique known as twin screw melt granulation (TSMG), which presents an alternative to traditional batch processing for granule production [29,30]. In this continuous process, a polymer, ideally with a low glass transition temperature (T_g_), serves as the binder instead of a granulating fluid [31]. When heated above its T_g_, the polymer melts and evenly coats the particles, forming granules [32]. Additionally, this method is capable of producing amorphous granules, which have the potential to enhance the solubility and bioavailability of drugs with poor water solubility [33,34].

The selection of the right polymer for the drug formulation is critical, particularly when considering water solubility. It has been demonstrated that using poorly water-soluble polymers can significantly limit drug release, resulting in an inadequate level of supersaturation. On the other hand, opting for polymers with high aqueous solubility may lead to issues with miscibility and hydrophobic interactions with poorly soluble drugs [35]. Balancing these factors highlights the complexity of selecting the right polymer at the right proportion for designing the solid dispersion.

In this context, miscibility refers to a molecular-level mixing of an amorphous system formed by combining an amorphous polymer with an amorphous API [36]. Melting point depression and lower enthalpy values have proved to be reliable indicators for anticipating drug–polymer miscibility, reflecting potential interactions between the components of the system [35,37,38].

Four model polymers were explored in this study: polyvinylpyrrolidone—Kollidon^®^ K12 PF (K12), polyvinylpyrrolidone vinyl acetate—Kollidon^®^ VA64 (KVA), polyvinyl caprolactam-polyvinyl acetate-polyethylene glycol graft copolymer—Soluplus^®^ (SOP), and Macrogol poly (vinyl alcohol) Grafted Copolymer—Kollicoat^®^ IR (KIR). Each pair of polymers was combined in equal parts to form binary blends, which were evaluated for their miscibility with simvastatin as well as their impact on solubility and drug release. The rationale for selecting these specific polymers lies in their diverse physicochemical properties, such as their amorphous or semicrystalline structure, hydrophilic or amphiphilic nature, and distinct glass transition temperatures. These characteristics provide a wide range of interactions with the drug. For instance, K12 and KVA as amorphous polymers are particularly effective in maintaining drug stability by preventing crystallization. SOP, due to its amphiphilic nature, enables the formation of micelles that encapsulate hydrophobic drug molecules, thereby enhancing their solubilization in aqueous environments [39,40]. KIR is a highly soluble polymer that can rapidly disintegrate in aqueous media. Its immediate-release properties complement the other polymers by ensuring rapid drug release and dissolution [41,42]. By exploring these polymer blends, we aimed to understand the synergistic effects these blends might have and identify the most promising formulations for enhancing the therapeutic efficacy of simvastatin.

In conclusion, the primary objective of this study was to evaluate the feasibility of utilizing hot-melt extrusion and twin-screw melt granulation as continuous manufacturing methods for producing simvastatin extrudates and granules in addition to comparing their release profiles to a marketed formulation. Furthermore, the study investigated how the use of polymer blends, whether miscible or immiscible with the drug, affects the solubility and release profile of simvastatin.

## 2. Materials and Methods

### 2.1. Materials

Simvastatin (purity 99.9%) was kindly gifted by BASF. PVP VA64 (Kollidon^®^ VA64), PVP K12 (Kollidon^®^ K12), Soluplus^®^, and Kollicoat IR^®^ (KIR, Macrogol poly (vinyl alcohol)) Grafted Copolymer were also generously gifted by BASF (Ludwigshafen, Germany). High-performance liquid chromatography (HPLC)-grade acetonitrile, sodium phosphate monobasic, and sodium hydroxide were obtained from Fisher Scientific. Deionized water (DI) and Milli-Q water were used throughout the study. Generic simvastatin tablets by Lupin Pharmaceuticals, Inc. (Baltimore, MD, USA) were used for comparative dissolution studies. The generic is approved by the FDA (ANDA 078103), assigned a therapeutic equivalence (TE) code of “AB”, and includes the following excipients according to the labeling [43]: ascorbic acid, citric acid, hydroxy propyl cellulose, hypromellose, iron oxides, lactose monohydrate, magnesium stearate, microcrystalline cellulose, pregelatinized starch, talc, titanium dioxide, and butylated hydroxyanisole

### 2.2. Drug–Polymer Miscibility by Modulated Differential Scanning Calorimetry (M-DSC)

M-DSC was performed for the accurate determination of the onset and enthalpy of the fusion of crystalline API alone and in the presence of the polymer blend to assess the miscibility of the API–polymer system. A sample of 3–5 mg of the PM (30% and 50% drug load) was equilibrated at 25 °C. M-DSC parameters were modulated at 1 °C every 60 s with heating rates of 2 °C/min from 25 °C to 160 °C. The Trios software (version 2.11.0.729) was used to examine the thermograms.

### 2.3. Equilibrium Solubility

An excess amount of crystalline SIM was added to 10 mL of 1% *w*/*w* binary polymer solutions (1:1) in a scintillation vial with a cap. The vials were shaken at 100 rpm for 24 h while being kept at 25 °C ± 0.5 °C in a glass shaker incubator. A Fisher Accuspin Micro 17 (Langenselbold, Germany) was used for the samples’ centrifugation at 13,000 rpm for 15 min, and the supernatants were analyzed using HPLC (*n* = 3). The reported data represent the averages of three measurements and their standard deviations.

### 2.4. Thermogravimetric Analysis (TGA)

A TGA 55 (TA Instruments, New Castle, DE, USA) was used to examine the thermal stability of pure SIM and the polymers. The experiment was conducted to determine the degradation onset temperature of SIM and the polymers and to optimize the extrusion temperature and residence time in the barrel. A high-temperature platinum open crucible containing (10 to 20 mg) of the samples was used for the TGA scans. The temperature was raised from 25 °C to 500 °C at a rate of 10 °C/min. The purge gas, ultra-purified nitrogen, was employed at a sample purge of 60 mL/min and a balance purge of 40 mL/min. Data collection and analysis was performed via Trios software (version 2.11.0.729).

### 2.5. Preparation of Physical Mixtures (PMs)

Table 2 outlines the compositions of all the formulations used to develop the solid dispersions, which include SIM at 30% *w*/*w* and a 1:1 ratio of polymeric combinations. The adequate quantities of the ingredients were weighed to prepare 35 gm batch sizes. Initially, the mixtures were blended by geometric mixing on wax paper, then mixed in a V-cone blender (Maxi-blend^®^, Globe Pharma, NJ, USA) for 10 min at 25 rpm. The batches were immediately processed after preparation.

### 2.6. Hot-Melt Extrusion and Twin Screw Melt Granulation

Initially, all the formulations were extruded using an 11 mm co-rotating twin screw extruder (Thermo Fisher Scientific, Waltham, MA, USA). Each batch was manually introduced into the feeding zone. The processing temperature was set to 150 °C across all zones, except for Zone Two, which was maintained at 25 °C to reduce polymer melting and prevent build up in the feeding zone. Torque did not exceed 30–35 Nm due to the relatively high temperatures employed across the zones. The extruder operated at a speed of 50 rpm, and had a standard screw configuration with three kneading zones and four conveying zones. The collected extrudates of all the formulations were milled using a benchtop blender, sifted through a #40 ASTM sieve, and stored in an airtight polybag until further characterization.

Subsequently, three formulations of interest with the highest and lowest miscibility and solubility were selected for granulation using the same extruder. A modified screw configuration was implemented, featuring one kneading zone comprised of four 90° elements and four 60° elements located between the third and fourth zones and maintained at 150 °C. Zones Five, Six, Seven, and Eight were set at temperatures of 50 °C, 50 °C, 25 °C, and 25 °C, respectively. Towards the end of the granulation screws, chopping elements were used to reduce the oversized fraction and achieve a more uniform particle size. The extruder operated at a speed of 50 rpm. The die plate at the end of the extruder barrel was removed to accommodate the granulation process and facilitate the granules’ collection.

### 2.7. Differential Scanning Calorimetry (DSC)

The physical state of the drug in the granules and the pure ingredients were further examined using a differential scanning calorimetry (DSC) system (TA Instruments, New Castle, DE, USA). The flow rate of nitrogen was maintained at 50 mL/min. Samples weighing 3–5 mg were placed in aluminum crucibles, sealed tightly with hermetic lids, and placed on the testing zone against a reference pan. After equilibration for one minute at 25 °C, the samples were heated to 200 °C at a rate of 10 °C/min.

### 2.8. Powder X-Ray Diffraction Analysis (PXRD)

The crystallinity of the pure SIM, polymers, physical mixtures, and granules was investigated using Powder X-Ray Diffraction (PXRD) analysis. Diffraction patterns were recorded with a Rigaku X-ray system (D/MAX-2500PC, Rigaku Corp., Tokyo, Japan) using Cu rays (λ = 1.54056 Å) at 40 kV and 15 mA. The scanning range was set from a 5 to 40 2-theta° scanning range, with a step width of 0.01°/s and a scan speed of 10 deg/min.

### 2.9. Scanning Electron Microscope (SEM)

The surface morphology of the pure drug, physical mixtures, and granules was explored using a JSM-7200FLV Scanning Electron Microscope (JEOL, Peabody, MA, USA) operating at an accelerating voltage of 10 kV. Samples were initially affixed to SEM stubs with double-sided adhesive tape. Prior to imaging, the samples were coated with a thin layer of platinum in an argon environment with a fully automated Denton Desk V TSC Sputter Coater (Denton Vacuum, Moorestown, NJ, USA).

### 2.10. Fourier Transform Infrared Spectroscopy (FTIR)

FTIR was conducted to study the interactions between SIM and the excipients in both the PMs and the extruded granules. The interactions depend on the molecular-level changes and involve the shifting or disappearance of the functional characteristic bands. The spectra of pure SIM, polymers, PMs, and their respective granules were analyzed using FTIR (Agilent Technologies Cary 660, Santa Clara, CA, USA). A small amount of sample was placed onto a diamond crystal and compressed using a Miracle high-pressure clamp. The bench was equipped with an ATR accessory (Pike Technologies MIRacle ATR, Madison, WI, USA) featuring a single-bounce diamond-coated ZnSe internal reflection element. Spectral scanning was performed over a range of 400–4000 cm^−1^ with a resolution of 4 cm^−1^.

### 2.11. High Performance Liquid Chromatography (HPLC)

The USP NF monograph HPLC method for the assay of SIM pure compound was used [44]. For the mobile phase, sodium phosphate monobasic buffer was adjusted to pH 4.5 using 0.2 N sodium hydroxide and combined with acetonitrile in a 35%: 65% (*v*/*v*) ratio, flowing at 1 mL/min. A 20 μL SIM injection was analyzed at a wavelength of 238 nm. The chromatographic system consisted of a Waters HPLC-UV system (Waters Corporation, Milford, MA, USA) equipped with a Luna C18 column from Phenomenex with dimensions of 150 mm length, 4.6 internal diameter, and 5 μm particle size. Stock solutions of SIM were prepared, mobile phase was used as a solvent, and five calibration standards were made, with concentrations ranging from 0.001 to 0.1 mg/mL. Linearity was evaluated by standard solution preparations. The retention time was approximately 4.9 min, with a total run time of 7 min. Regression analysis of the calibration curve resulted in an R^2^ value of 0.9999%.

### 2.12. In Vitro Release Studies

Dissolution studies of SIM formulations were conducted using the extrudates, granules, and marketed tablets. The milled extrudates and granules were passed through a US# 40 mesh sieve. A quantity equivalent to 20 mg SIM was encapsulated in size 00 gelatin capsules and placed in sinkers. The release study was carried out in a USP type II dissolution apparatus, Erweka, (Korsh–Erweka GmbH, Heusenstamm, Germany) at 37 °C ± 0.5 °C, at 50 rpm, and with 900 mL of monobasic sodium phosphate buffer adjusted to pH 7, with sodium hydroxide and 0.2% sodium dodecyl sulphate (SDS) to maintain sink conditions. Aliquots were withdrawn at specified time intervals of (5, 10, 15, 30, 45, and 60 min) centrifuged at 13,000 rpm for 15 min using a Fisher Accuspin Micro 17 (Langenselbold, Germany), and the supernatant was analyzed for SIM content using the area under the curve and the regression equation derived from the aforementioned HPLC method. The withdrawn samples were replaced immediately with equal amounts of fresh and preheated dissolution medium. As per the USP NF dissolution method, the acceptance criterion is not less than 75% of simvastatin dissolved in 30 min [44].

### 2.13. Statistical Analysis

The in vitro release profiles of granules, PMs, and the pure drug were presented as mean values accompanied by standard deviations (SD). Statistical analysis was conducted by comparing the release profile of granules with that of the PMs and the pure drug using Student’s *t*-test with Prism software (Version 8.3.0). The significance level was set at α = 0.05. For comparing the release profile of the extrudates and the granules to the marketed formulation, an *f*2 test was used, and the mathematical formula is given below.

(1) $$f_{2}=50\times log\left\{{\left[1+\left(1∕n\right)\sum_{j=1}^{n}{\left|R_{j}-T_{j}\right|}^{2}\right]}^{-0.5}\times 100\right\},$$

where $f_{2}$ is the similarity factor used in comparing two dissolution profiles, *n* is the number of time points, *Rj* and *Tj* are the reference and test dissolution values at the rth and j-th time point, respectively.

## 3. Results and Discussion

### 3.1. Drug–Polymer Combinations Miscibility

The M-DSC analysis was conducted to precisely determine the drug’s onset melting temperature and enthalpy values and to differentiate between reversible and non-reversible events [45]. Initially PMs of 50% *w*/*w* SIM and polymer blends were prepared to have a clear estimation of these values since melting peaks are expected to be more emphasized at higher drug concentration. Furthermore, the results were confirmed with 30% *w*/*w* drug–polymer ternary systems. Table 3 outlines the peak temperature, onset temperature and enthalpy values for the neat crystalline SIM, and the polymeric blends at 30% and 50% *w*/*w* drug load.

**Figure 1:**
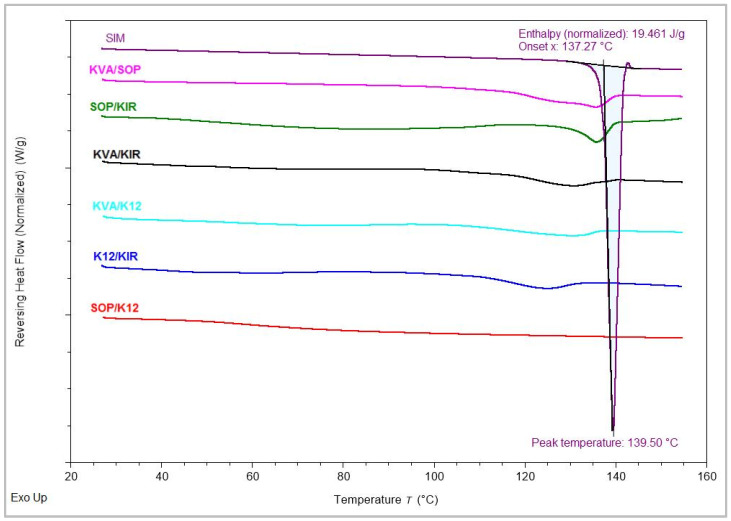
M-DSC analysis for SIM and the PMs.

The M-DSC thermogram of SIM operated at lower speed of 2 °C/min is characterized by an endothermal process that corresponds to its melting peak at 139.50 °C and enthalpy value of 19.5 J/g. At a 50% *w*/*w* drug load, the results indicate that drug–polymer ternary systems containing the K12 polymer exhibited a greater reduction in SIM melting temperature and enthalpy values less than 5 J/g, implying enhanced miscibility with polymer combinations including K12. Specifically, SOP/K12, KVA/K12, and K12/KIR had onset temperatures of 115.6, 114.3, and 111.8 and enthalpy values of 4.5, 3.7, and 3.2, respectively. The blend with K12/KIR had a relatively lower enthalpy value compared to the individual blend of SIM and K12, which had an onset temperature of 111.7 °C and an enthalpy value of 4.08 J/g. On the other hand, the rest of the polymer combinations displayed reduced the melting point depression and higher enthalpy values, which is indicative of lower miscibility. At a 30% *w*/*w* drug load, the formulations demonstrated a consistent miscibility pattern, despite minor variations. The order from highest to lowest miscibility according to enthalpy values was SOP/K12, followed by K12/KIR, KVA/KIR, KVA/K12, SOP/KIR, and KVA/SOP. According to the melting point depression and enthalpy values, it can be concluded that the systems comprising K12/KIR and SOP/K12 were highly miscible with SIM, while KVA/SOP demonstrated the lowest miscibility among all the polymeric combinations. Figure 1 graphically represents the M-DSC analysis of the 30% *w*/*w* ternary polymer systems, clearly highlighting the differences in melting point depression among the various blends.

### 3.2. Drug–Polymer Combinations Aqueous Solubility

**Figure 2:**
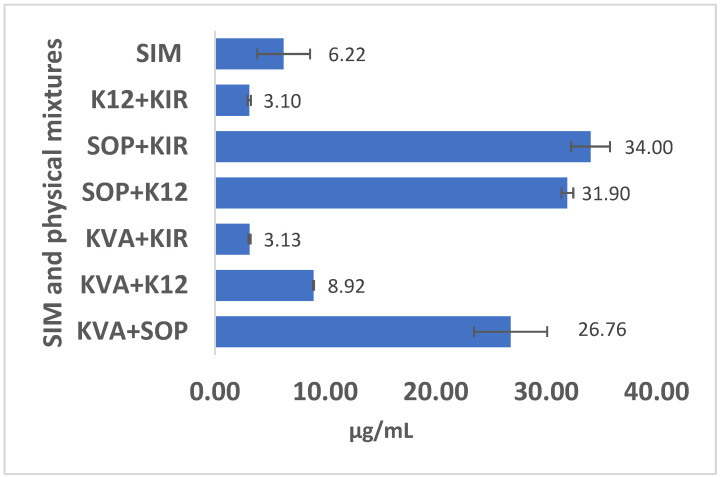
Equilibrium solubility of SIM in water and 1% polymer solutions.

The solubility of the neat crystalline SIM in water was found to be 6.22 ± 2.39 µg/mL. The measured solubility values of SIM in 1% polymeric solutions are presented in Figure 2, highlighting the varying solubilizing efficiencies of the polymeric combinations used. The results demonstrated higher solubility for the 1% polymeric solution including Soluplus^®^ (SOP) and following the order SOP/KIR > SOP/K12 > SOP/KVA. This enhanced solubility is attributed to Soluplus’^®^ amphiphilic nature as a copolymer composed of polyvinyl caprolactam, polyvinyl acetate, and polyethylene glycol. Its bifunctional properties enable micelle formation and improve the dissolution rate of hydrophobic drugs while maintaining supersaturation throughout the gastrointestinal tract [4,7]. It is worth mentioning that the Hansen solubility parameter difference between SIM and SOP, as predicted using Zoomlab^TM^, was 3.7 MPa^1/2^. The total solubility parameter of a molecule reflects its ability to interact based on contributions from dispersion forces, polar interactions, and hydrogen bonding contributions. Typically, a solubility parameter difference less than 7 MPa^1/2^ is indicative of high miscibility between SIM and SOP.

Notably, the SOP/KIR combination exhibited a substantial improvement in solubility, achieving approximately 34 µg/mL ± 1.75 dissolved SIM, which represents a 5.5-fold increase compared to the aqueous solution of SIM alone. Conversely, the simvastatin solubility rate was remarkably lower in the K12/KIR polymeric combination, despite the high miscibility observed in the DSC analysis. The solubility of SIM in this blend was lower than that of the pure drug; however, the difference was not statistically significant (*p* = 0.14). Similarly, the solubility of SIM in the KVA/KIR combination was also low, with no significant difference compared to the pure drug (*p* = 0.16). The reduced solubility in these blends could be due to the high viscosity of the polymer blend, which might hinder the dissolution of SIM in water. Another possible reason could be a complexation between the drug and the polymers, which may have reduced the availability of free SIM for dissolution.

In summary, the analysis of miscibility and solubility results revealed that the combinations of miscible API and polymers do not always translate into high API solubilizing capacity. This phenomenon indicates a nuanced interplay between miscibility and solubility enhancement mechanisms. While miscibility reflects uniform distribution of the drug within the polymer matrix, increased miscibility does not necessarily correlate with increased solubility of the API.

Similarly, a research study exploring the relationship between the drug–polymer miscibility and solubility of posaconazole (POS) and four different polymers demonstrated that the systems with the highest miscibility tended to exhibit lower solubility, while those with lower miscibility showed higher solubility. Particularly, POS had high miscibility with KVA and Kollidon K30, less miscibility with KIR, and was immiscible with Parteck MXP (PXP, polyvinyl alcohol); however, the highest solubility was obtained with the POS + PXP system [35].

### 3.3. Thermogravimetric Analysis

Prior to performing a melt extrusion process, it is critical to confirm the thermal stability of the drug and excipients. Experimental data plotted the percentage weight loss of SIM and the polymers against the temperature. SIM exhibited signs of degradation starting at 220 °C, with a 10% weight loss recorded by 260 °C. About 80% of the drug is lost between 220 °C and 290 °C, with a second phase of degradation that started above 300 °C, and the results are similar to the published results [46,47]. KVA showed an initial mass loss attributable to absorbed moisture below 100 °C, while degradation started at temperatures above 250 °C. Similarly, SOP and K12 demonstrated moisture-driven mass loss below 100 °C, while their degradation onset occurred at temperatures exceeding 300 °C [48]. Also, KIR demonstrated weight loss and degradation signs above 200 °C [19]. These results indicate that SIM and the studied polymers are stable under the extrusion temperatures of 150 °C.

### 3.4. Hot-Melt Extrusion

Preliminary M-DSC studies were conducted for drug loadings ranging from 30% to 70%. The results demonstrated that a 30% drug loading exhibited reduced enthalpy levels, indicating a higher likelihood of successful amorphization of the drug in the polymer blends during extrusion. Furthermore, this drug loading was identified as optimal for achieving smooth and efficient processing.

**Figure 3:**
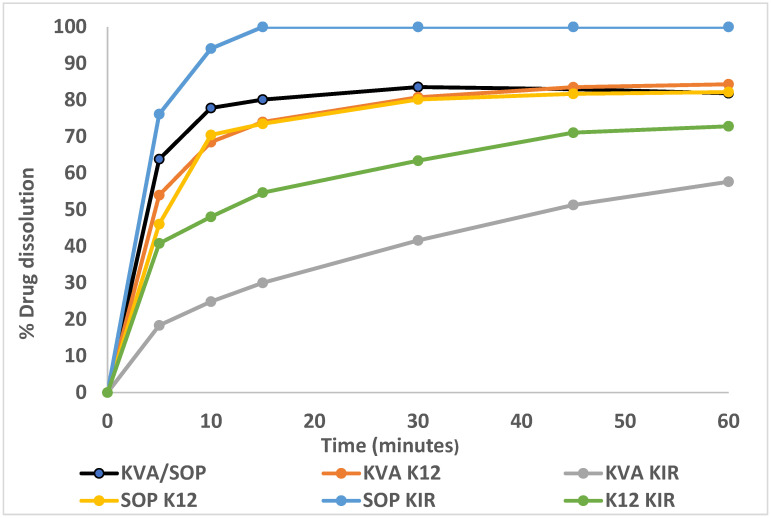
SIM dissolution from the SD extrudates after 10, 30, and 60 min.

Following the extrusion process, the resulting extrudates from all the formulations were utilized for subsequent in vitro release studies to investigate their release behavior relative to the solubility study results. The initial release study was performed using extrudates in a phosphate buffer of pH 7 with 0.5% *w*/*v* SDS at 37 °C ± 0.5 °C and 50 rpm, as recommended by the USP NF monograph. However, the dissolution medium was indiscriminatory, and no significant differences were observed between the formulations. Thus, dissolution studies were conducted using the same conditions with 0.2% *w*/*v* SDS instead of 0.5% *w*/*v*. Figure 3 represents findings from these dissolution studies at different time intervals that illustrate very rapid release onset of the SOP/KIR polymer combination that exceeds 85% in 15 min and a relatively slower release from the KVA/KIR and K12/KIR combinations. These findings were in line with the equilibrium solubility studies. Based on these results, formulations of interest were selected for twin screw melt granulation processing.

### 3.5. Twin Screw Melt Granulation (TSMG)

Based on the findings from the miscibility, solubility, and dissolution studies, three formulations (F1, F3, and F6) were selected for further granulation processing. KVA/SOP (F1), identified as the least miscible, exhibited the highest enthalpy values of (11.31 J/g) and (3.39 J/g) at 30% and 50% *w*/*w* drug loads, respectively. The formulations of SOP/K12 (F2) and K12/KIR (F6) were highly miscible with SIM, recording enthalpy values of 4.52 and 3.22 J/g at 50% *w*/*w* drug load and 0.89 and 2.05 J/g at 30% *w*/*w*/drug load, respectively. The K12/KIR (F6) results were particularly intriguing as they demonstrated lower enthalpy values at higher drug loading. Additionally, this combination exhibited the lowest aqueous solubility and relatively slow in vitro release. These characteristics make K12/KIR (F6) worthy of further investigation. According to the solubility results, SOP/KIR (F3) exhibited the highest solubility, dissolving approximately 34 μg/mL of SIM in 1% polymer solution, in addition to its rapid release of more than 90% in 10 min. Conversely, K12/KIR (F6) showed a SIM solubility of 3.10 μg/mL and a release of less than 40% in 10 min and less than 60% in 60 min. Accordingly, further granulation was pursued with F1, F3, and F6 formulations.

**Figure 4:**
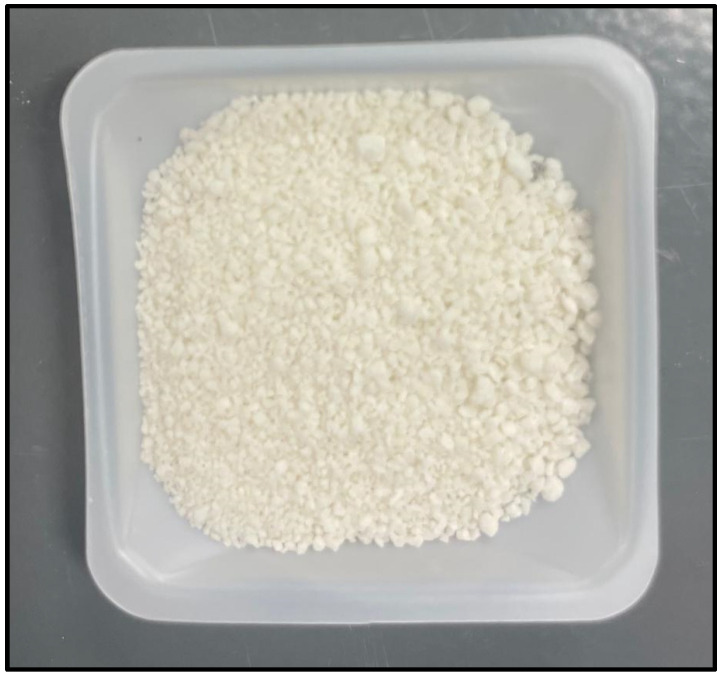
Developed granules with SOP/KIR blend using TSMG.

For successful granulation, the outlet zones need to be maintained at a temperature below the T_g_ of the polymers. Thus, the initial granulation trials using the standard screw configuration employed for extrusion led to excessive torque values and extruder shutdown. To address this, a modified screw configuration with one kneading zone was implemented to mitigate the excessive stress. The modification proved successful, with torque values recorded between 30 to 33 N.m. The kneading zone located at Zone Three and the next one were maintained at a temperature of 150 °C. Lower temperatures resulted in higher torque levels and inconsistent feeding. Meanwhile, the temperatures in Zones Five to Eight were kept low at 50 °C, 50 °C, 25 °C, and 25 °C, respectively, to cool down the granules and facilitate their formation. The screw speed was set at 50 rpm to ensure adequate blending of materials and to reduce the crystallinity of SIM. The image of the granules from the SOP/KIR formulation is shown in Figure 4.

### 3.6. Thermal Analysis (DSC)

**Figure 5:**
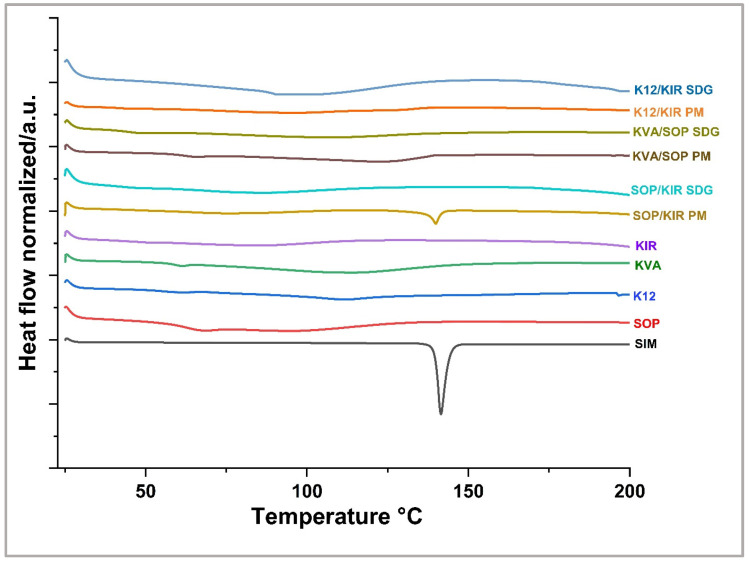
DSC thermograms for SIM, polymers, selected SDGs, and PMs.

The DSC heating curves depicted in Figure 5 demonstrate the thermal behavior of SIM, the polymers, and the SDGs alongside their corresponding PMs. Crystalline SIM is characterized by an endothermal process marked by its melting peak at 140 °C and enthalpy of fusion value of 73.5 J/g. SOP, PVP, and PVA represent amorphous polymers, as evidenced by the absence of endothermic events in their thermograms. The broad peak observed before 100 °C corresponds to moisture loss upon heating. As reported in the literature, the polymers’ glass transition temperatures (T_g_) were observed around 70 °C, with 90 °C and 111 °C for SOP, K12, and KVA, respectively [48,49,50]. On the other hand, KIR is a semi-crystalline polymer characterized by the grafting of polyethylene glycol and polyvinyl alcohol, displaying a T_g_ at about 45 °C and a melting peak at 208 °C [51,52].

The thermogram of the physical mixtures exhibited a subtle SIM endothermic peak, which is masked by the polymer-wide peaks. However, this peak is visible for the SOP/KIR PM due to its higher enthalpy value. Analysis of the SDGs thermograms revealed the absence of SIM peaks for all the formulations. This finding implied the amorphization of SIM within the binary polymeric blend under the employed extrusion temperature. Attempts of extrusion at a temperature below 150 °C resulted in partially crystalline formulations, as confirmed by DSC.

### 3.7. PXRD Analysis 

**Figure 6:**
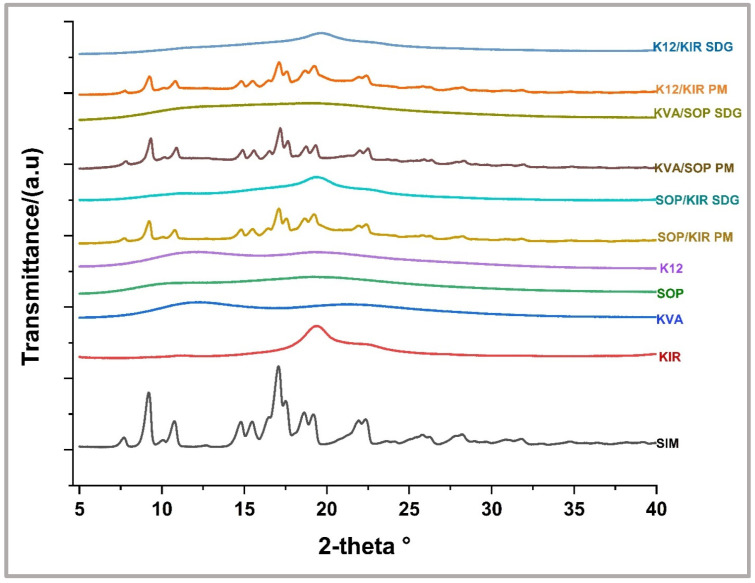
PXRD for SIM, polymers, selected SDGs, and PMs.

The XRD analysis of SIM and the individual polymers is illustrated in Figure 6. The diffractogram of pure SIM exhibits distinct peaks observed at 2θ values of 9.26°, 10.75°, 17.03°, 17.47°, 18.62°, 19.20°, and 22.44°, indicative of its crystalline nature [13]. Similarly, the diffractograms of the physical mixtures retained these characteristic peaks of the drug, with a noticeable reduction in the peaks’ intensity. In contrast, the granules displayed a broad halo, signifying the disappearance of all the peaks. This XRD data complements the findings from the DSC analysis and confirms the transformation of the crystalline drug into its amorphous form upon interaction with the polymers during the granulation process.

### 3.8. SEM Analysis 

**Figure 7:**
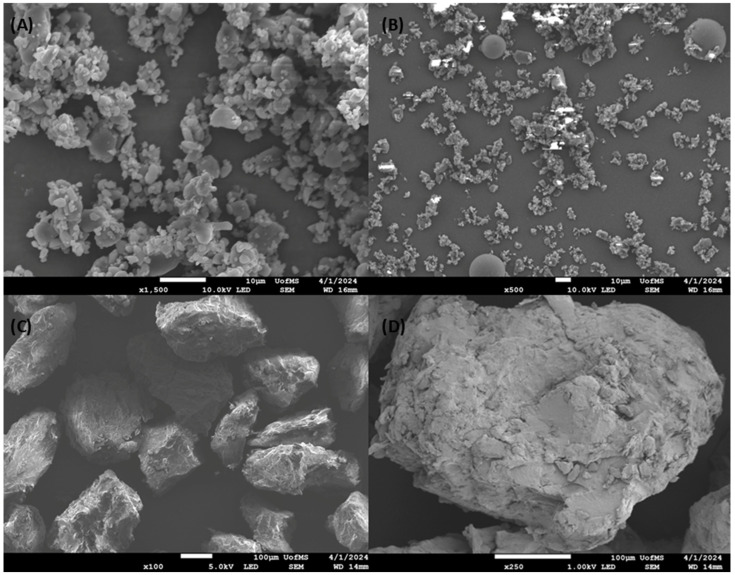
SEM images showing surface morphology of (**A**) simvastatin crystals (×1500), (**B**) SIM-K12/KIR PM (×500), and (**C**) (×100), (**D**) (×250) SIM-K12/KIR granules.

The SEM images in Figure 7 provide insights into the morphology of SIM, PM, and the granules. Figure 7A highlights the irregularly sized cylindrical crystals of pure SIM with a clear tendency to aggregate. In contrast, the physical mixture shown in Figure 7B reveals a heterogeneous composition, with visible clustered SIM crystals surrounded by spherical polymer particles, confirming the absence of homogenous mixing at the molecular level.

The granules depicted in Figure 7C,D exhibit large, dense, and porous agglomerates, consistent with the polymer encapsulation of SIM during the granulation process. Notably, the absence of discrete agglomerated crystals on the surface or background supports the hypothesis of the thorough dispersion of SIM within the polymer matrix. These SEM observations align with DSC and XRD results, which confirm the transition of SIM into an amorphous state, providing robust evidence of its successful incorporation into the granules.

### 3.9. FTIR Analysis

**Figure 8:**
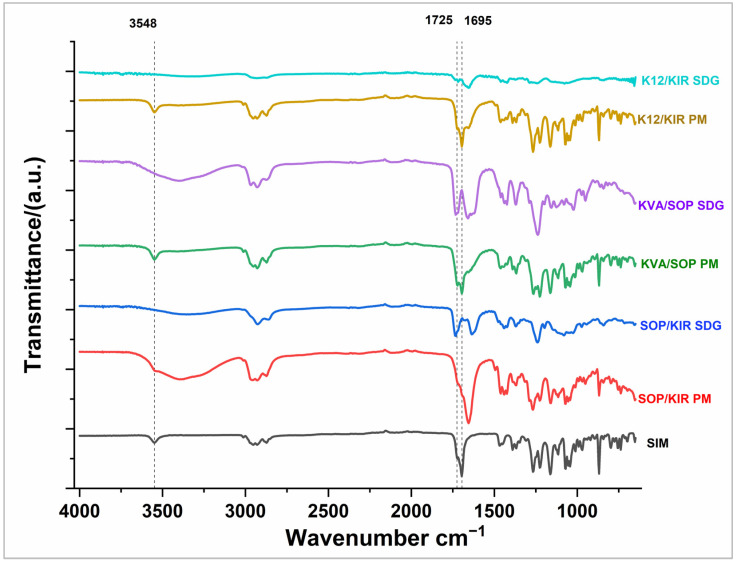
FTIR for SIM, PMS, and selected SDGs.

The infrared spectra of the individual polymers are outlined in Table 4. The spectra of the pure drug, PMs, and granules are displayed in Figure 8. Analysis of the IR spectrum of the pure drug reveals characteristic bands at specific wavenumbers. The band at 3548 cm^−1^ indicated the presence of (hydroxyl−OH group stretching vibration), 3011.6 cm^−1^ for (aromatic C=CH stretching vibration), 1725 cm^−1^ (stretching vibration for aliphatic ester C=O), 1695 cm^−1^ (stretching vibration for lactone C=O), 1468, 1390, and 1367 cm^−1^ (methyl and methylene bending vibration), and 1263, 1222, 1162, and 1114 cm^−1^ corresponding to (lactone and ester −C−O−C− bending vibration).

It is observed from the figure that the characteristic OH stretching band of SIM at 3548 cm^−1^ is preserved in the physical mixtures, indicating no significant molecular interactions between SIM and the polymers in these mixtures. However, this band was broadened in all the granules, suggesting that the hydroxyl group of SIM has engaged in hydrogen bonding with polar groups, such as the carbonyl groups of SOP, K12, and KVA.

Similarly, the carbonyl stretching vibrations for SIM aliphatic ester (1725 cm⁻^1^) and lactone (1695 cm⁻^1^) are clearly visible in the physical mixtures but are significantly diminished or masked in the spectra of the SOP/KIR and KVA/SOP granules. This suggests that the carbonyl groups of SIM shifted to shorter wavenumbers, likely due to hydrogen bonding. The split and intensified carbonyl bands observed in SOP-based formulations are attributed to overlapping and intensification caused by potential hydrogen bonding with the terminal hydroxyl groups of SOP, and the hydroxyl groups in the PVA backbone of KIR. Furthermore, the absence of the sharp SIM crystalline bands in the granules further supports the hypothesis that SIM has been strongly engaged in intermolecular interactions, likely forming hydrogen bonds with the polymer matrix.

Particularly noteworthy is the substantial reduction in band intensity observed with the K12/KIR combination, suggesting significant molecular interactions of the drug with this polymer blend likely due to hydrogen bond formation, which yields a more homogenous mixture. This observation aligns closely with the M-DSC results, showing lower enthalpy values for this combination, which further confirms the strong interactions and enhanced miscibility between SIM and K12/KIR.

The FTIR results collectively demonstrate favorable interactions between the drug and polymer matrix, primarily through hydrogen bonding, which play a key role in stabilizing the system. The amorphous nature of the drug within the solid dispersion is particularly advantageous as it typically enhances dissolution rates. Additionally, the surrounding polymer matrix provides a stabilizing environment that prevents recrystallization, ensuring sustained solubility and dissolution performance.

### 3.10. Release Studies

**Figure 9:**
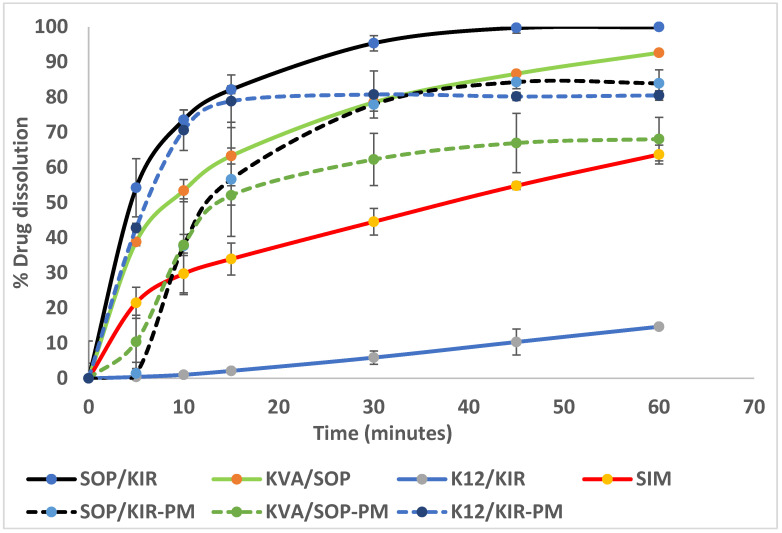
Dissolution profiles of crystalline SIM, PMS, and SDGs at 37 °C ± 0.5 °C in pH 7 phosphate buffer using 0.2% *w*/*v* SDS.

An in vitro release study was performed using the modified USP method for SIM tablets previously discussed. Granules (SDGs) weighing 66.67 mg, corresponding to 20 mg of SIM, were placed in 900 mL of pH 7 phosphate buffer with 0.2% *w*/*v* SDS at 37 °C ± 0.5 °C, and the medium was stirred with a paddle set at 50 rpm. The release profiles of crystalline SIM, the physical mixtures (PMs), and the granules are presented in Figure 9. The dissolution rates for the extrudates discussed previously were slightly faster, with more SIM released from the extrudates than the granules over the study period (Figure 3). This can be attributed to the screw configuration and higher extrusion temperature, which enhanced the intimate mixing of the drug with the polymer. This improved dispersion ensures the drug is more readily available for dissolution. Additionally, the higher thermal energy applied during extrusion compared to granulation likely contributed to improved dissolution characteristics. Previous research demonstrated the impact of different screw configuration on the release profiles of the solid dispersions and the physical state of the API [53,54,55].

Merely using the PMs resulted in an enhancement in drug dissolution compared to the crystalline form, which is attributed to the influence of hydrophilic polymers. For the SOP/KIR PM, the release reached approximately 80% within 30 min. Similarly, the K12/KIR PM also achieved around 80% release in the same timeframe. In contrast, the KVA/SOP PM demonstrated a lower release rate, reaching 62% after 30 min. Subsequently, drug release continued to rise over 60 min, reaching approximately 84% for SOP/KIR PM, 68% for KVA/SOP PM, and plateauing at 80% for K12/KIR PM.

On the other hand, SDG had a rapid dissolution rate from the highly soluble polymeric combination of SOP/KIR that reached 82.1% ± 2.8% in 15 min, 95.3% ± 1.10 in 30 min, and 99.7 ± 1.53% in 45 min. The reason for the enhanced dissolution is the overall enhanced hydrophilicity of the system, with the PEG component present in both SOP and KIR potentially acting as a good solubilizer of SIM, combined with the conversion of the API to the amorphous form and its entrapment within the polymer blend. It is well known that the amorphous form has fewer thermodynamic barriers for dissolution due to the absence of the crystalline lattice and the higher molecular motion and internal energy, which they quickly release by forming hydrogen bonds with solvents, facilitating dissolution. Additionally, the random arrangement of atoms in amorphous forms allows solvents to easily penetrate and dissolve the solid particles [56,57]. The dissolution results correlate well with the DSC and PXRD studies, which confirm the amorphization of the API.

Conversely, the release from K12/KIR granules exhibited an unexpectedly slow rate compared to its PM. This may be due to complexation between SIM and the polymer combination during the granulation process. The heightened miscibility confirmed by thermal analysis and FTIR suggests strong interactions between SIM and the polymers, which likely contributed to the formation of a viscous gel-like structure during dissolution and SIM molecule diffusivity entrapment, which implies the entrapment of drug molecules within the polymer matrix. In contrast, the PM has less pronounced interactions, leaving the drug more accessible during dissolution.

The release from the SOP/KIR granules demonstrates the superior performance of the drug release rate and extent compared to other SIM solid dispersions reported in previous research studies. For instance, a solid dispersion developed through supercritical carbon dioxide, employing Soluplus as the main polymer and 30% drug load, had an approximately 90% release rate in 60 min under the same dissolution conditions [4]. Another SIM solid dispersion prepared by co-evaporation with croscarmellose sodium and sodium starch glycolate released 100% of SIM in 90 min and 93% in 120 min, respectively, under the same dissolution conditions and higher amount of SDS (0.5% SDS) [58]. Another study developed simvastatin (SIM) solid dispersions using hydroxypropyl methyl cellulose (HPMC) grades E3 and E5 through HME and spray drying techniques. For a 20% drug load, the release from HME with HPMC E3 was 79.2%, and with HPMC E5, it was 70.5% in 60 min. In comparison, the spray-dried formulations with the same drug load showed a release of 86.21% with HPMC E3 and 78.5% with HPMC E5 in 60 min under identical dissolution conditions with 0.5% SDS [17].

**Figure 10:**
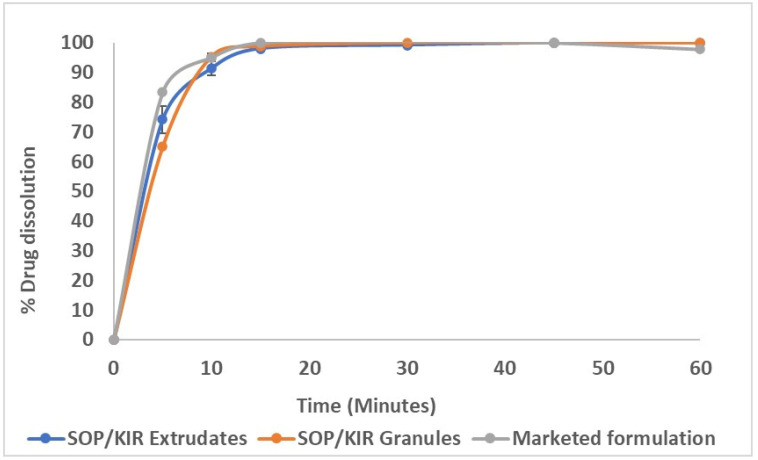
In vitro release profiles of SOP/KIR granules and extrudates versus the marketed formulation at 37 °C ± 0.5 °C in pH 7 phosphate buffer using 0.2% *w*/*v* SDS.

The in vitro release of SIM from the marketed tablet formulation (Simvastatin by Lupin Pharmaceuticals, 20 mg) was compared to that of the SOP/KIR granules and extrudates, each containing an equivalent amount of SIM (20 mg), as illustrated in Figure 10. The dissolution profiles of the extrudates, the granules, and the marketed formulation were similar, with *f*2 values of 51.7 for the granules and 64.4 for the extrudates. Both granules and extrudates demonstrated a very rapid dissolution, achieving over 85% release within 15 min. This result is higher than the acceptance criterion of not less than 75% of simvastatin dissolved in 30 min, even with a lower concentration of SDS (0.2%) in the dissolution medium. The improved dissolution of SIM in the marketed formulation could have been due to the presence of multiple excipients, including HPC and hypromellose, and perhaps the manufacturing method played an additional role.

In summary, this study explored various polymer combinations and their interactions with simvastatin, focusing on their impact on solubility and dissolution enhancement. While the miscibility results obtained from M-DSC provided valuable insights, they could not fully explain the observed improvements in solubility and dissolution rates. For instance, the highly miscible K12/KIR system did not translate into improved solubility or dissolution. A possible explanation is that the enhanced miscibility demonstrated by thermal analysis and FTIR indicates strong interactions between SIM and the polymers, which led to the formation of a viscous gel-like structure during dissolution. This structure may trap SIM molecules within the polymer matrix, thus reducing their diffusivity.

The mechanism behind the superior performance of the SOP/KIR blend could be attributed to their unique structural properties, since both SOP and KIR contain polyethylene glycol (PEG) monomers, which are well known for enhancing hydrophilicity and promoting the solubilization of hydrophobic drugs. Another contributing mechanism could be the presence of hydroxyl groups—terminal hydroxyl groups in SOP and hydroxyl groups within the PVA backbone of KIR. These hydroxyl groups can form additional hydrogen bonds with the carbonyl groups of SIM, strengthening drug–polymer interactions and potentially stabilizing SIM in its amorphous state.

Future work could benefit from advanced studies such as molecular dynamics simulations to identify the specific monomers or functional groups responsible for interactions with SIM, which could provide a detailed understanding of the intricate mechanisms driving solubility and dissolution enhancement. Additionally, a Flory–Huggins interaction parameter analysis could offer further insights into the molecular-level interactions and thermodynamic compatibility between the drug and the polymers. Expanding this work to include stability studies and in vivo testing would also validate the clinical relevance of these findings.

## 4. Conclusions

This study focused on evaluating the effectiveness of TSMG in enhancing the solubility and dissolution profile of SIM, a poorly water-soluble drug. Notably, SIM-SOP/KIR, which was both extruded and granulated, showed the highest SIM solubility, whereas SIM-K12/KIR had the lowest. The extrusion and the granulation processes were performed using an 11 mm co-rotating twin screw extruder. Characterization of the resulting SDGs through DSC and PXRD confirmed the transformation of SIM from its crystalline form to an amorphous state across all formulations. This amorphization is a critical factor in enhancing the solubility and dissolution rates of crystalline APIs. Dissolution studies revealed significantly improved rates for the SIM-SOP/KIR system compared to the physical mixture (PM) and the pure drug, with both SIM-SOP/KIR extrudates and SDGs displaying rapid dissolution profiles similar to the commercially marketed tablet formulation. Future studies will evaluate the stability of the SDGs and its correlation with the miscibility data, investigate the tableting potential of the granules, and assess the impact of compression and additional excipients on the dissolution rate of the final product. Overall, our findings underscore the potential of TSMG as an efficient single-step continuous manufacturing technique for producing granules with enhanced solubility and dissolution profiles. This study also emphasizes the complexity of polymer–drug interactions and the critical importance of selecting complementary polymers to effectively improve the solubility and dissolution of poorly water-soluble drugs.

## Figures and Tables

**Table 1:** Examples of SIM SD prepared by various techniques reported in the literature.

Technique	Polymers, Excipients	Reference
Kneading and solvent evaporation	PEG 6000 and PVP K30	[12]
Spray drying	PVP K25 and Aerosil 200	[13]
Supercritical carbon dioxide	Soluplus	[4]
Quench cooling	PVP K12	[14]
Fusion and solvent evaporation	HPMC and gum acacia	[15]
Physical trituration, kneading, and solvent evaporation	PEG 8000, PVP K30, and SLS	[16]
HME and spray drying	HPMC E3 LV and HPMC E5 LV	[17]
Supercritical antisolvent process	Hydroxy propyl β-cyclodextrin	[18]
Solvent evaporation method	Ternary SD of PEG 12,000 and a surfactant (Pluronic F68, Pluronic F127, SLS, or Myrj 52)	[19]
Fusion and solvent evaporation method	Xylitol, sorbitol, Soluplus, and lactulose	[20]

**Table 2:** Composition of the drug–polymer ternary systems.

Formulation	SIM	SOP	KVA	K12	KIR
F1	30%	35%	35%	-	-
F2	30%	35%	-	35%	-
F3	30%	35%	-	-	35%
F4	30%	-	35%	35%	-
F5	30%	-	35%	-	35%
F6	30%	-		35%	35%

**Table 3:** Thermal properties of the physical mixtures.

Formulations		50% *w*/*w* SIM			30% *w*/*w* SIM		
	Sample	Peak Temp.	Onset Temp.	Enthalpy	Peak Temp.	Onset Temp.	Enthalpy
		°C		J/g	°C		J/g
	SIM	139.50	137.27	19.5	139.50	137.28	19.5
PM 1	SIM-KVA/SOP	136.99	131.90	11.31	117.49	135.68	3.39
PM 2	SIM-SOP/K12	128.54	115.61	4.52	107.24	119.64	0.89
PM 3	SIM-SOP/KIR	138.48	135.3	9.21	135.80	130.50	2.48
PM 4	SIM-KVA/K12	125.80	114.28	3.66	108.79	129.67	2.3
PM 5	SIM-KVA/KIR	135.94	126.8	8.02	117.99	129.89	2.15
PM 6	SIM-K12/KIR	122.92	111.84	3.22	111.78	123.89	2.05

**Table 4:** ATR–FTIR peak table of all the polymers.

Ingredient	Description	Wavenumber (cm^−1^)
Kollidon 12PF (K12)	O–H stretching of absorbed water	3429
	C=O–stretching vibration	1654
Soluplus (SOP)	aromatic C–H stretching	2925
	C=O stretching	1733–1625
	C–O–C stretching	1478
Kollicoat IR (KIR)	aliphatic hydroxyl group	3280
	CO stretching band	1084
Polyvinylpyrrolidone (KVA) Vinyl acetate	vinyl acetate group	1733
	vinylpyrrolidone group	1669

## Data Availability

The data presented in this study are available within the article.
